# Asynchronous Changes in Vegetation, Runoff and Erosion in the Nile River Watershed during the Holocene

**DOI:** 10.1371/journal.pone.0115958

**Published:** 2014-12-31

**Authors:** Cécile L. Blanchet, Martin Frank, Stefan Schouten

**Affiliations:** 1 GEOMAR Helmholtz Centre for Ocean Research Kiel, Kiel, Germany; 2 Department of Marine Organic Biogeochemistry, NIOZ-Royal Netherlands Institute for Sea Research, ‘t Horntje (Texel), The Netherlands; Institute of Botany, China

## Abstract

The termination of the African Humid Period in northeastern Africa during the early Holocene was marked by the southward migration of the rain belt and the disappearance of the Green Sahara. This interval of drastic environmental changes was also marked by the initiation of food production by North African hunter-gatherer populations and thus provides critical information on human-environment relationships. However, existing records of regional climatic and environmental changes exhibit large differences in timing and modes of the wet/dry transition at the end of the African Humid Period. Here we present independent records of changes in river runoff, vegetation and erosion in the Nile River watershed during the Holocene obtained from a unique sedimentary sequence on the Nile River fan using organic and inorganic proxy data. This high-resolution reconstruction allows to examine the phase relationship between the changes of these three parameters and provides a detailed picture of the environmental conditions during the Paleolithic/Neolithic transition. The data show that river runoff decreased gradually during the wet/arid transition at the end of the AHP whereas rapid shifts of vegetation and erosion occurred earlier between 8.7 and ∼6 ka BP. These asynchronous changes are compared to other regional records and provide new insights into the threshold responses of the environment to climatic changes. Our record demonstrates that the degradation of the environment in northeastern Africa was more abrupt and occurred earlier than previously thought and may have accelerated the process of domestication in order to secure sustainable food resources for the Neolithic African populations.

## Introduction

The Sahara Desert is presently one of the most arid regions on Earth, but also experienced some of the most drastic environmental changes of the past 10,000 years. During the African Humid Period (AHP, ∼10,000 to ∼6,000 calendar years before present, hereafter referred to as ka BP), monsoonal rains reached areas of the Sahara Desert much further north than today and permitted the development of grasslands and water bodies that hosted large herds of wild game [1]. Human populations thrived in this fertile environment and occupied a large part of the nowadays hyper-arid desert [2]. Due to decreasing summer insolation in the intertropical zone (i.e., between 20°S and 20°N), the rain belt and the vegetation receded to the South and forced the human populations to leave the Sahara at ∼6.5 ka BP [2], [3]. It was during this period of large environmental changes that major societal reorganizations occurred, such as the implementation of pastoralism and agriculture into human lives, as well as the development of collective rituals and religious beliefs [3], [4]. Uncontroversial archeological evidence demonstrates the presence of domesticated cattle in human settlements from ∼8 ka BP onwards in northern Egypt (Nabta) [5]–[7]. There is an ongoing debate as to whether North Africa has been a center of domestication or whether the domesticated cattle found there originated from the Near East [7]. In any case, North Africa played a decisive role as a focal center and a corridor for the dispersal of domesticates throughout the African continent [6]. Furthermore, the initiation of food production in NE Africa occurred with a delay of about 2000 years as compared to the Fertile Crescent of the Near East and exhibited a very distinctive pattern. Archeological evidence in NE Africa suggests that animal domestication occurred without agriculture but within mobile hunter-gatherer groups and post-dated the development of pottery [8]. The role of the major local environmental changes and their influence on these different models of food production has not yet been clearly determined.

The environmental degradation at the end of the AHP has been considered an important factor for the introduction of domesticates in Neolithic populations [4]. However, the exact timing of the wet-dry transition in NE Africa is still debated. A reconstruction of environmental changes obtained from geological and archeological data showed a gradual decline in rainfall and a southward retreat of the vegetation during the Holocene [2]. Such a gradual transition was also observed in the pollen and sedimentological record of Lake Yoa (central Sahara) for the past 6 kyr [9] and in the speleothem record of paleo-rainfall on the Oman Peninsula between 8 and 2 ka BP [10]. This view has recently been challenged by the reconstruction of the wet-dry transition within in a few centuries around 5.5 ka BP from a sediment core off Somalia, similar to records from western Africa [11]. These discrepancies question the exact role of environment-human relationships at the beginning of the Holocene.

**Figure 1:**
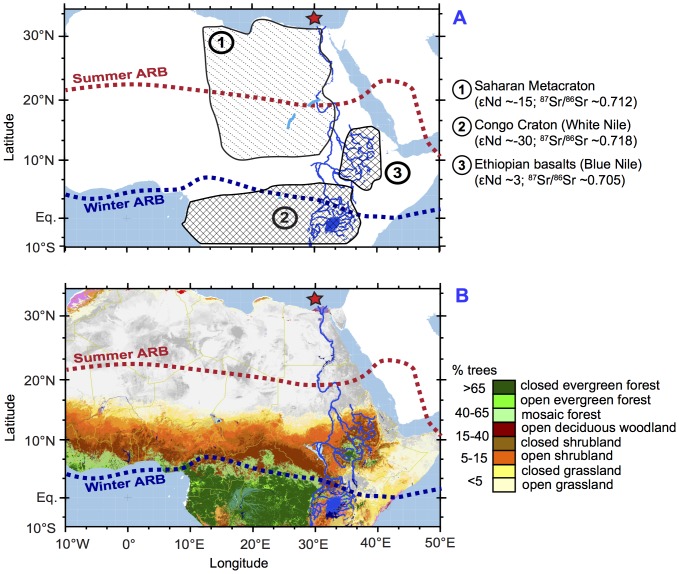
Map of the present-day land cover in northern Africa. A: Location and averaged radiogenic isotope composition of the three main sources of sediments to the Nile deep-sea fan [44]. B: Types of vegetation and estimated percentage of trees at present in Northern Africa [58]. The gray color represents the Sahara Desert, which is presently non-vegetated. The course of the Nile River is represented by the dark blue line. The location of core P362/2-33 is indicated by the red star. The present-day northern reach of the summer and winter African Rain Belt (ARB) are depicted as red and blue dashed lines, respectively.

Here we present an alternative view on the evolution and the timing of environmental changes during the Holocene by reconstructing conjointly the changes in vegetation, erosion and rainfall dynamics from the same sedimentary archive. This allows the establishment of a precise timeframe for the different processes in order to understand their causal relationships and provides a detailed environmental context for human evolution in North Africa. We used sediment core P362/2-33 that was retrieved from the Nile deep-sea fan at 700 m water depth [12], [13] (Fig. 1). The unique feature of this 6-m long core is a 5-m thick section of finely laminated sediments, which were deposited during the AHP and which are a local expression of sapropel S1 [13]. These sediments offer a very high temporal resolution of several mm/year and provide an integrated record of past environmental changes within the Nile watershed. During the AHP, the drainage area of the Nile was significantly larger than today due to the contribution from lake and river systems located in the present-day Sahara Desert [1], [14]. The fluvial input of particulate and dissolved matter that has been stored in sediments on the Nile deep-sea fan is thus a unique source of information on past environmental changes in NE Africa.

## Material and Methods

Marine sediment core P362-2/33 (700 m water-depth, 31°40.51N; 29°45.00E) was retrieved during the project “West Nile Delta” funded by RWE-Dea but not specifically for the purpose of the present study. The cruise permit 201–2008 was issued on the 21.01.2008 by the Egyptian Ministry of Defense (Authority of Armed Forces Operations, through the Naval Forces based at Alexandria Naval Base) for RWE-Dea. The core location is in public area. No human or vertebrate animals were used during this study.

The sedimentation on the Nile deep-sea fan is largely controlled by the seasonal discharge of the Nile River (especially at the location of core P362/2-33, which is in the vicinity of the most active Rosetta Canyon) [12]. Precipitation patterns at the source of the Nile River largely determine sediment provenance: While the White Nile and Sobat River contribute a minor part of the annual sediment load (3.5%). but provide a quasi-constant flow of water to the Nile, the Blue Nile and Atbara River provide the major part of the annual sediment load (96.5%) mainly during boreal summer (Fig. 1a) [15]. Eolian dust originating from North African sources and hemipelagic sedimentation (diatoms, foraminifera) also contribute to sedimentation at the core location during the spring and winter months [13].

### Description and age control of sediment core P362/2-33

A detailed description of core P362/2-33 and the age model were published previously in ref. [13]. The upper 70 cm of the 6-m long sediment core consist of brownish to greyish bioturbated sediments, below which faint laminations are observed between 75 and ∼105 cm core depth. Well-preserved millimeter-scale laminations are observed below 140 cm core depth and most probably result from deposition of highly dense, suspension-rich (hyperpycnal) flows formed by particle-laden seasonal Nile flood plumes in the water column [16]. The age model of the sediment core was based on fourteen ^14^C ages of fossil planktonic foraminifera shells, measured at the Leibniz-Laboratory for Radiometric Dating and Isotope Research (University of Kiel). Correction of reservoir ages and calibration of the ^14^C ages are described in ref. [13]. The core covers most of the Holocene, with the bottom of the core being dated at ∼9000 ^14^C yrs BP (9.5 ka BP) and the uppermost sediment sections were deposited prior to 1800AD. Sedimentation rates vary from ∼650 cm/kyr in the lower part of the core to ∼8 cm/kyr in the upper part.

### Stable oxygen isotope of foraminifera

Stable oxygen isotopes (δ^18^O_C_) were measured every 3 cm on the planktonic foraminifera *G. ruber* (white and >250 µm) at the GEOMAR (n = 176) (S1 Table). The sediment samples were washed and dry-sieved and ∼20 specimens were picked, crushed and gently cleaned with ultrapure (Milli-Q) water. The shells were dissolved in orthophosphoric acid at 70°C in a Kiel II carbonate device and the CO_2_ released was analyzed by a Thermo Scientific MAT 252 isotope ratio mass spectrometer (IRMS). The ^18^O/^16^O ratios are reported in ‰ relative to the Vienna-Pee Dee Belemnite (VPBD) standard, where: δ^18^O = [((^18^O/^16^O)_sample_/(^18^O/^16^O)_VPDB_)-1]*1000. Analytical precision of <0.06 ‰ for the δ^18^O measurements was determined from repeated analyses of a Solnhofen limestone which is calibrated against NBS19 as internal standard.

### Radiogenic neodymium and strontium isotope signature of detrital sediments

The methodology used for extracting neodymium (Nd) and strontium (Sr) signatures from detrital sediments is similar to that described in ref. [13]. However, the set of samples published in ref. [13] (a few grain-size fractions) is very different to the one we present here (bulk sediment measurements for the whole core), which is unpublished. The bulk sediment samples (∼3 g) were first decarbonated (using a buffered acetic acid solution, pH ∼4.5), then leached to remove any authigenic ferro-manganese coatings [17] (using a buffered hydroxylamine hydrochloride solution, pH ∼3.9) and finally ∼0.05 g of the homogenized detrital sediment was totally dissolved (using Aqua Regia, HNO_3_ conc. and HF conc.) (S2 Table). The samples were purified and separated using standard ion-chromatography procedures [18], [19]. The Nd and Sr isotope compositions were measured on a Nu Instruments multi-collector inductively-coupled plasma mass spectrometer at GEOMAR. Blank concentrations were negligible for isotopic analyses (<0.3 ng for Nd and <3,4 ng for Sr).

External reproducibility (2σ) was first estimated by repeated measurements of in-house SPC and SPEX standards for Nd and the AA standard for Srranging from ±0.14 to ±0.63 εNd units (±14–63 ppm, n = 57) for Nd and from ±0.000009 to ±0.000048 (±12–68 ppm, n = 58) for Sr. External reproducibility was further assessed by repeated measurements of the JNdi standard for Nd isotopes and the NBS SRM 987 standard for Sr isotopes, and yielded 2σ uncertainties of ±0.3 εNd units (±30 ppm, n = 57) and ±0.00003 (±42 ppm, n = 58) for Sr. The isotope results reported were normalized to the accepted values of the JNdi standard for Nd (^143^Nd/^144^Nd = 0.512115) and of the NBS SRM 987 standard for Sr (^86^Sr/^87^Sr = 0.710245). The Nd isotope ratios are reported as εNd: 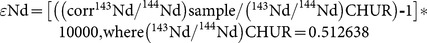 [20].

### Alkenones, stable carbon isotopes of n-alkanes and BIT index

The lipids were extracted from 43 sediment samples with a DIONEX Accelerated Solvent Extractor 200 at the NIOZ using a solvent mixture of 9∶1 (v/v) dichloromethane (DCM)/methanol (MeOH). After the addition of internal standards C_22_ anti-isoalkane (*n-*alkanes), 10-nonadecanone (alkenones) and C_46_ glycerol trialkyl glycerol tetraether (GDGTs), the total lipid extract was separated into apolar, ketone and polar fractions using pipette column chromatography loaded with aluminum oxide and the solvent mixtures 9∶1 (v/v) hexane/DCM, 1∶1 hexane/DCM and 1∶1 DCM/MeOH as eluents, respectively. The apolar fraction was then separated into saturated hydrocarbon (long-chain odd *n-*alkanes and the C_22_ anti-iso standard) and aromatic fractions using pipette columns loaded with Ag^+^-impregnated silica and hexane and ethylacetate as eluents, respectively.

Molecular identification of the alkenones and *n-*alkanes was performed on a Thermo Finnigan Trace Gas Chromatograph (GC) Ultra coupled to a Thermo Finnigan DSQ mass spectrometer (MS). The alkenones and the *n*-alkanes were quantified using HP 6890 GCs with CP Sil-5 columns (50 m for the alkenones and 25 m for the *n-*alkanes) and helium as the carrier gas. The Uk_37_′ index [21], defined as C_37∶2_/(C_37∶2_+C_37∶3_), was used to estimate surface seawater temperatures (SSTs) for 39 samples run in duplicate (mean reproducibility of ±0.8°C) (S4 Table and S1b Fig.), following the equation [22], [23]: SST = −0.957+54.293(Uk_37_′)−52.894(Uk_37_′)^2^+28.321(Uk_37_′)^3^.

The stable carbon isotope compositions of the long-chain odd *n-*alkanes were measured for 41 samples (in duplicate or triplicate) on an Agilent 6800 GC coupled to a ThermoFisher Delta V Isotope Ratio MS (S3 Table and S2 Fig.). Isotope values were measured against calibrated external reference gas and the performance was checked daily by injection of two calibrated *n-*C_20_ and *n-*C_24_ perdeuterated *n-*alkane standards. The ^13^C/^12^C isotope ratios of *n-*alkanes are reported in the standard delta notation (δ^13^C) in ‰ against the V-PDB standard: δ^13^C = [((^13^C/^12^C)_sample_/(^13^C/^12^C)_VPDB_)-1]*1000. The average reproducibility is 0.47‰ for the *n-*C_27_ *n-*alkane, 0.32‰ for *n-*C_29_, 0.29‰ for *n-*C_31_ and 0.49‰ for *n-*C_33_. The average reproducibility of the internal C_22_ anti-iso standard was 0.9‰ (n = 62) and the reproducibility of an external *n-*C_24_ standard was 0.46% (n = 31). A weighted average was calculated using the relative proportion (area under the peak divided by the sum of the areas of *n*-C_27_, *n*-C_29_, *n*-C_31_ and *n-*C_33_) and isotope ratio of each *n-*alkane (S2 Fig.). The estimation of the % fraction of C_4_ plants (%C_4_) was realized using a two end-members mixing equation [24]: %C_4_ = 100-(−7.4627* δ^13^C_wax_−160.82). The terrestrial provenance of the *n-*alkanes was assessed by calculating the carbon preference index (CPI), which is the ratio between odd and even *n-*alkanes [25]. Long-chain odd *n-*alkanes originate from terrestrial higher plants with a CPI of >3, whereas long-chain even *n-*alkanes originate from petroleum sources with a CPI of ∼1. The CPI in core P362/2-33 ranges from 3 to 9 with a mean value of 6.6, which indicates the terrestrial origin of the *n-*alkanes throughout the record.

The polar fraction of 41 samples (S4 Table), containing the GDGTs, was dissolved in a mixture of 99∶1 (v/v) hexane/propanol and filtered through 0.45 mm PFTE filters. GDGTs were analyzed (in triplicate) by high performance liquid chromatography (HPLC)/MS in single ion monitoring mode on an Agilent 1100 series LC/MSD SL [26], [27]. The Branched and Isoprenoid Tetraether (BIT) index was calculated following [28]: BIT =  (GDGT-I+GDGT-II+GDGT-III)/(GDGT-I+GDGT-II+GDGT-III+Crenarchaeol), whereby the GDGTs refer to structures shown in S3 Fig. The averaged standard deviation for the calculated BIT index is ±0.01.

### Results and interpretations

**Figure 2:**
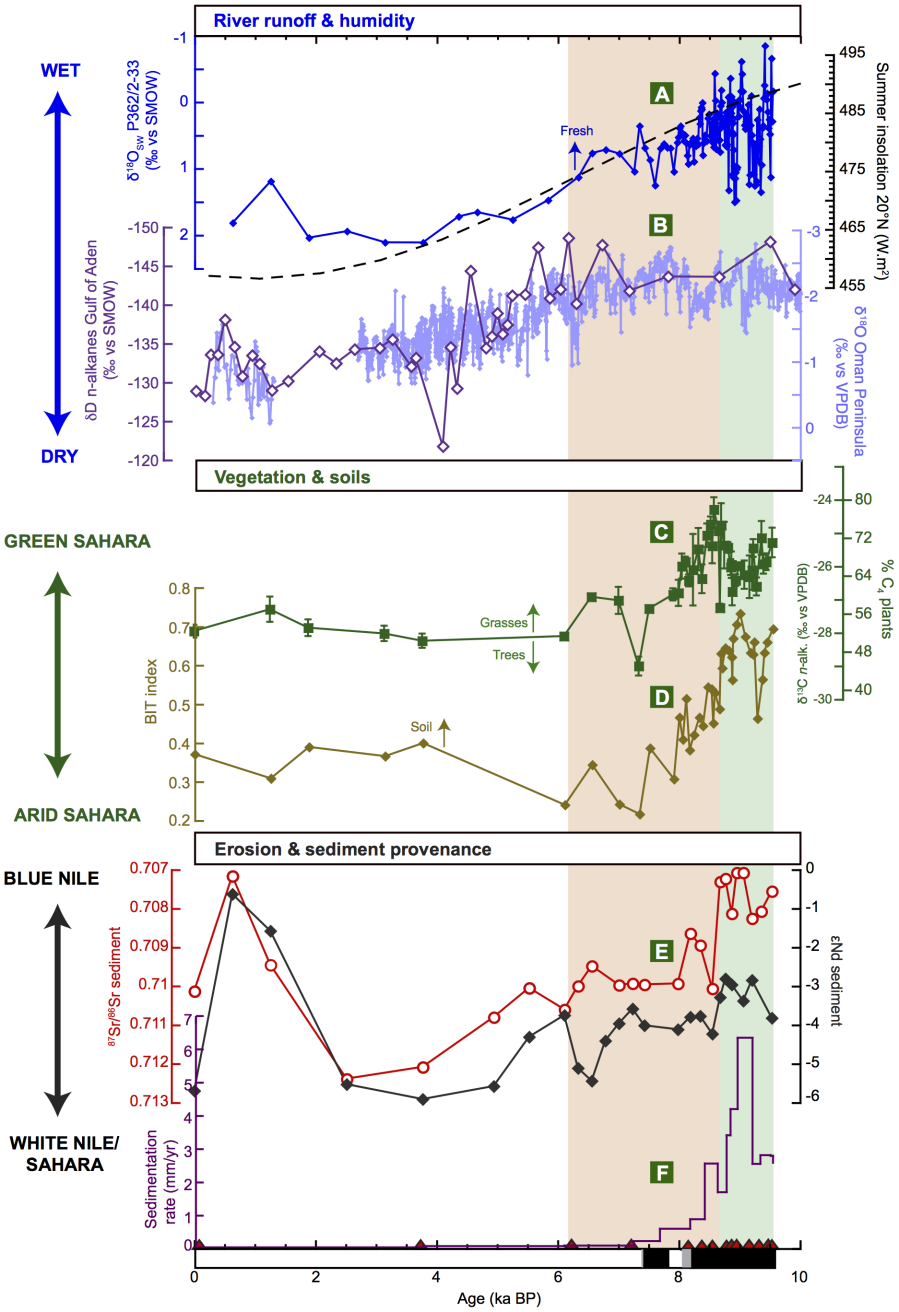
Changes in precipitation, vegetation and erosion dynamics in the Nile watershed during the Holocene. A: Summer (June-August) insolation at 20°N [59] (dashed line) and oxygen isotope signature of the surface seawater (δ^18^O_SW_) at the location of our core, which reflects changes in sea-surface salinity and river runoff (S1 Fig.) and has been controlled by orbitally-induced changes in precipitation. B:. Paleo-precipitation records obtained from a speleothem on the Oman Peninsula (δ^18^O), rainfall regime of which has been under influence of the Indian monsoon system [10] and obtained from marine sediments off the coast of Somalia (δD of n-alkanes), the rainfall regimes of which has been under the influence of deep-convection in the Indian Ocean [11]. C: Stable carbon isotope composition of higher-plant n-alkanes reflecting the proportion of C_4_ (mainly grasses) versus C_3_ plants (trees/shrubs). D: BIT index recording changes in the relative contribution of soil organic matter input. E: Radiogenic Sr and Nd isotope signatures of the bulk detrital fraction of the sediments documenting changes in sediment provenance. F: Sedimentation rates. Radiocarbon dates are indicated at the bottom of the panel as red triangles and black rectangles at the bottom of the figure indicate the laminated parts of the core (see “Material and Methods” section and ref. [13]).

The strength of river runoff is reconstructed from stable oxygen isotope compositions of surface seawater obtained from planktonic foraminifera (δ^18^O_SW_) (Fig. 2a). The δ^18^O_SW_ record was obtained by correcting the measured δ^ 8^O signature of planktonic foraminifera for changes in surface seawater temperature (SST) determined using the U^k^′_37_ paleothermometer (S1 Fig.). The SSTs reconstructed for core P362/2-33 fall in the same range as those previously reconstructed in the Levantine Basin (16 to 26°C) (S1b Fig.) [29], [30]. To estimate the δ^18^O_SW_, we used the paleotemperature equation established for *Orbulina universa* [31] and applied a correction for changes in the ice volume [32]. The δ^18^O_SW_ values obtained for Late Holocene sediments are similar to the present-day eastern Mediterranean seawater δ^18^O [33] (S1c Fig.) and the values for the Holocene fall in a similar range and follow a trend similar to those previously obtained in the Levantine Basin (∼2 to −1‰) [29]. This demonstrates that the reconstructed δ^18^O_SW_ is not biased by the fact that foraminifera and coccolithophores bloom during different seasons (summer and spring, respectively) and therefore potentially recorded different SSTs (see discussion in ref. [29]). The δ^18^O_SW_ has primarily been related to the changes in surface seawater salinity (SSS) changes, which has been influenced by the precipitation/evaporation balance and by the changes in river runoff (amount effect) [34]. However, as recently shown [35], the δ^18^O_SW_/SSS relationship may have varied during the Holocene due to changes in the δ^18^O of freshwater end-member (i.e., the precipitation and the Nile River water), resulting from changes in the hydrological cycle or in the provenance of the river water. In our case, these various effects cannot be deciphered but they all have the same effect on the δ^18^O_SW_: a decrease in δ^18^O_SW_ can be interpreted as a decrease in salinity (either due to a higher precipitation/evaporation ratio or increased runoff), and/or as an increase in δ^18^O of the freshwater end-member (either due to a more intense convection or a dominant Blue Nile source for the river water [36], which both led to higher runoff). During the early Holocene, δ^18^O_SW_ values around 0‰ were closer to the present-day δ^18^O signature of Nile River water (0.8 to −0.6‰, ref. [36]) and might therefore reflect enhanced river runoff leading to lower salinities (S1c Fig.). During the early Holocene, the δ^18^O_SW_ exhibited large amplitude and high frequency variations, which might be related to a large variability of the Nile runoff during the so-called ‘Wild Nile’ period (Fig. 2a). The geomorphological record of this time interval has demonstrated the occurrence of very intense floods with Nile river levels up to 5 m higher than at present [15], [37]. This is also reflected by very high sedimentation rates and the deposition of mm-thick layers of flood-derived sediments in our record (Fig. 2f) [13].

The close similarity in long-term trends between our δ^18^O_SW_ record and the speleothem δ^18^O record of paleo-precipitation on the Oman Peninsula suggest that river runoff responded quasi-linearly to changes in rainfall intensity in the Nile watershed (Fig. 2b) [10]. The speleothem record of Quunf Cave on the Oman Peninsula exhibits a gradual decline in precipitation throughout the Holocene that followed the changes in summer insolation, which is also observed in our δ^18^O_SW_ record. This implies that non-linear and threshold mechanisms (such as lake overflow) played a limited role in controlling freshwater discharge. Recently, another paleoprecipitation record was obtained from δD analyses of n-alkanes in marine sediments retrieved off the coast of Somalia/Ethiopia that challenged the prevailing consensus of a gradual wet/dry transition at the end of the AHP in Eastern Africa [11]. Contrary to the speleothem record from the Oman Peninsula and other regional records [2], [9], [10], this δD record exhibits a rapid decrease in rainfall/humdity between ∼6 and 4 ka BP (Fig. 2b). The authors infer that such a rapid termination of the AHP (which was also observed in other records from eastern African lakes [11]) was due to the fact that the hydroclimate regime on the Horn of Africa has mostly been under the influence of deep convection and sea surface temperatures in the western Indian Ocean.

New environmental information on the evolution of vegetation cover, soil dynamics and erosion patterns has been generated from sediment core P362/2-33. Changes in vegetation cover are reconstructed using the stable carbon isotope composition of long-chain odd *n-*alkanes, which originate from higher plant leaf waxes (δ^13^C_wax_) (Fig. 2c). Plants with C_4_ and C_3_ photosynthetic pathways (essentially corresponding to warm-season grasses/sedges and cool-season grasses/trees/shrubs, respectively) produce leaf waxes with different δ^13^C values that are preserved during transport and sedimentation, and particularly during oxidative diagenesis [38]–[40]. Here we use the weighted average of δ^13^C of four *n-*alkanes (*n-*C_27_, *n-*C_29_, *n-*C_31_ and *n-*C_33_) to estimate changes in the contribution of plants using a C_4_ metabolic pathway to the *n*-alkane pool (%C_4_) in the drainage basin (Fig. 2c). Due to their CO_2_-concentrating mechanism, C_4_ plants (such as warm-season grasses and sedges) are generally isotopically enriched in ^13^C compared to C_3_ plants, which include most trees, cool-season grasses, and sedges [40]. The δ^13^C records measured on each *n-*alkanes have similar long-term trends, with higher values (reaching −21‰ for *n*-C_33_) between 9.5 and 8 ka BP and lower values (down to −30‰ for *n*-C_31_) between 6 and 0 ka BP (S2 Fig.). The estimation of the % fraction of C_4_ plants (% C_4_) was realized using the 2 end-members mixing equation from ref. [24] and the terrestrial provenance of the n-alkanes was assessed by calculating the Carbon Preference Index (see “Material and Methods”). The *n-*alkanes can be transported by wind or rivers but it is assumed here that they have been mainly transported to the core site by the Nile River because the concentration and accumulation rates of *n-*alkanes show a long-term trend similar to that of the branched GDGTs, which are transported only by rivers (S3a,b Fig.) [28]. Furthermore, Blanchet et al. [13] have shown that at core site P362/2-33, the terrigenous sedimentation has been largely controlled by fluvial inputs and that eolian inputs were significant only around 3–4 ka BP, when the amount of *n-*alkanes was low in the sediments (S3a Fig.). The δ^13^C_wax_ varied by 5‰ in our record, which corresponds to proportional abundances of C_4_ plants between ∼80 and 40% (Fig. 2c), although these numbers have to be interpreted with some care due to the widely varying concentrations of n-alkanes in C3 and C4 plants [41]. The prevalence of C_4_ grasses during the AHP reflects the northward migration of the African Rain Belt that led to the expansion of C_4_-dominated savannah-type vegetation into areas of the Sahara that are not vegetated today (Fig. 1b) [42], [43]. A rapid stepwise decrease in δ^13^C_wax_ between 8.5 and ∼7.8 ka BP and between 6.5 and 6 ka BP (from −24 to −28‰) documents a drastic reduction in C_4_ plant cover, which was a consequence of the retreat of the vegetation in the Sahara during the southward migration of the African Rain Belt at the end of the AHP.

Another prominent feature of our record is the abrupt switch in sediment provenance accompanied by a decrease in soil organic matter input and erosional activity. The amount of soil organic matter input to the sediments was estimated using the Branched and Isoprenoid Tetraethers (BIT) index (Fig. 2d), which is the ratio between the contents in terrigenous branched GDGTs and marine crenarchaeol [28]. Castañeda et al. [30] showed that the BIT index can be strongly influenced by the production of marine crenarchaeol and therefore advised to compare the contents and accumulation rates of branched GDGTs and crenarchaeol to the BIT index. At the site of core P362/2-33, the BIT index has obviously been affected by changes in the soil organic matter content rather than by the changes in crenarchaeol content (S3b,c,d Fig.), which suggests that the changes in the BIT index most likely reflect changes in soil formation and erosion. A marked decrease in soil organic matter input is documented by the BIT index from 0.7 to 0.2–0.3 between ∼9 and 7.3 ka BP (Fig. 2d). This decrease in soil organic matter input is nearly synchronous with a switch in sediment provenance recorded in the radiogenic Nd and Sr isotope signatures (εNd and ^87^Sr/^86^Sr) of the detrital sediment fraction (Fig. 2e). As shown on Fig. 1a, the sources of the Nile River are characterized by specific εNd and ^87^Sr/^86^Sr signatures depending on their lithology [44]. At 8.7 ka BP, the εNd and ^87^Sr/^86^Sr of the detrital sediment fraction recorded an abrupt shift from more basaltic (εNd ∼−3 and ^87^Sr/^86^Sr ∼0.707) to more granitic (εNd ∼−4 and ^87^Sr/^86^Sr ∼0.71) signatures. This documents a decrease in the proportion of sediments originating from the Blue Nile (Ethiopian Highlands) and an increased supply of sediments from the White Nile/Sahara regions (Fig. 2e). This enhanced contribution from the White Nile/Sahara regions was already reported in core P362-2/33 based on the grain-size distribution of the terrigenous fraction (i.e., changes in the proportion of grain-size end-members) [13]. This switch in sediment provenance was accompanied by a drop in sedimentation rate from 6 mm/yr to less than 2 mm/yr between 9 and 8.7 ka BP (Fig. 2f) and implies that the massive erosion of Blue Nile soils due to intense flooding activity decreased abruptly at 8.7 ka BP and thus preceded the rapid degradation of the green Sahara.

## Discussion

These drastic modifications of the environment during the early Holocene reconstructed from the proxy-record of core P362/2-33 have important implications. Firstly, the proxy-records of the termination of the AHP in NE Africa show considerable spatial and temporal variability. While some records have depicted a gradual and sometimes stepwise desiccation following the decrease in summer insolation [9], [45], [46], others indicate a rapid desiccation around 5.5 ka BP that occurred significantly faster than the change in orbital forcing [11], [47]. Our record supports an alternative scenario in which a rapid degradation of the environment occurred between 8.7 and 6 ka BP, significantly earlier than previously inferred from eastern Saharan records (Fig. 2). It must also be noted that this environmental degradation occurred while water discharge in the Nile drainage basin and precipitation on the Oman Peninsula [10] decreased gradually and did not exhibit a rapid wet/dry transition between 4 and 6 ka like on the Horn of Africa [11]. This non-linear response of the environment to changes in river discharge (precipitation) might be explained by feedback processes or threshold mechanisms between vegetation and precipitation [48], [49]. A recent study highlighted the potential role of differential responses of distinct components of the vegetation system during a climatic transition [50]. Depending on their sensitivity to environmental constraints, various types of plants exhibit distinct behavior during a climatic transition, but the presence a large diversity of plant types (including more resilient plants) might help to stabilize the environment and generate a gradual change. The pollen record of a gradual transition of vegetation during the AHP termination at Lake Yoa might reflect such a process [9]. In contrast, our record of an early abrupt retreat in grass cover might reflect the high sensitivity of C4 plants to small changes in precipitation. A modeling study described the process by which the vegetation system degraded quickly (faster than the forcing) after reaching a precipitation threshold, causing the switch to the other stable equilibrium condition, the desert state [49]. If this interpretation were correct, it would imply that large-scale vegetation changes might occur in the absence of strong precipitation-vegetation feedback processes (i.e., the degradation of the vegetation did not provoke a further decrease in precipitation) contrarily to what was proposed in earlier studies [48].

An early arid event such as that in our record at ∼8.7 ka BP was previously identified in several records in NE Africa around 8.6–8.2 ka BP as a transient episode that sometimes marked the initiation of the AHP termination [45]–[47], [51]. In our record, however, this event marked a permanent modification of erosion dynamics and vegetation cover. It was claimed previously that arid events in North Africa resulted from teleconnections to cooling events in the Atlantic (such as the 8.2 ka event) [3] given that they occurred during periods of low sea-surface temperatures in the Mediterranean [52] and the tropical Atlantic Ocean [53]. However, our record shows that the onset of desiccation in the Nile watershed preceded the cooling event by at least 500 years, which suggests that arid events in North Africa may as well have resulted from internal destabilization of the climate-vegetation system, which in turn may have influenced the climate at higher latitudes [46], [54].

Another important implication of our record is the establishment of a detailed environmental context for the major reorganizations within the human populations in the Sahara Desert. The transition between the Paleolithic and the Neolithic is marked by the beginning of food production by the human populations and coincided with the drastic modification of the environmental conditions in NE Africa. The beginning of food production occurred through animal domestication in the form of mobile herding [6], [8] but the origin of the domesticated cattle, whether it was introduced from the Near East or originated from a local bovid source remains an unresolved question [5], [7]. In any case, northern Africa has played a decisive role as a focal center and a corridor for the dispersal of domesticates throughout the African continent [6].

**Figure 3:**
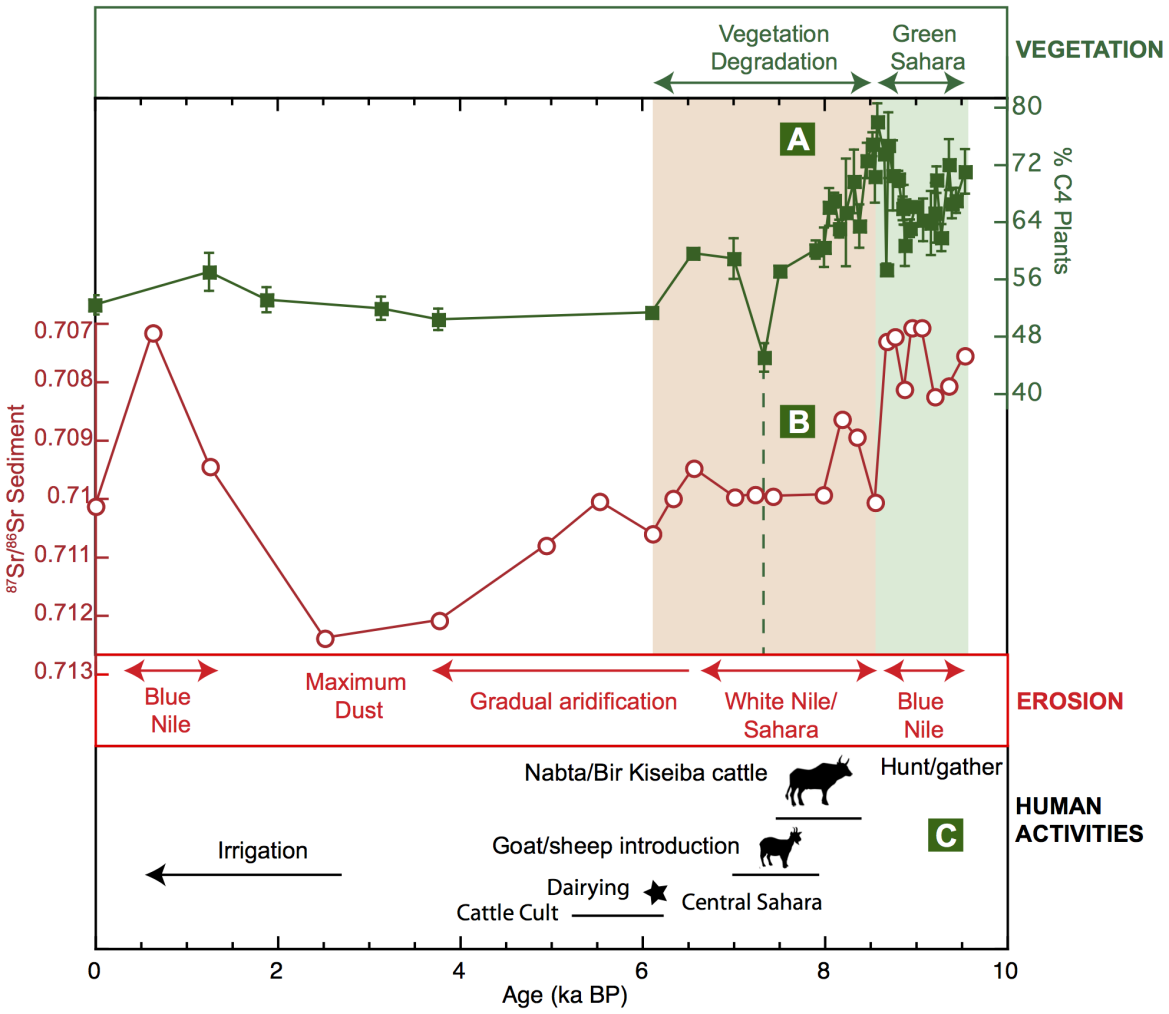
Environmental context of major steps of human evolution in NE Africa during the Holocene. A: Percentage of C4 plants, as estimated from the δ^13^C of higher-plants n-alkanes (see Fig. 2). B: Sediment source as estimated from the radiogenic Sr isotope signature of the detrital sediment fraction. C: Phases of human evolution, as compiled from ref. [5]–[8] and [54]–[56].

Uncontroversial archeological remains date the first domesticated cattle at around 8.5–7.5 ka BP in the Nabta-Bir Kiseiba region [5]–[7] immediately following the significant environmental change that occurred in the Nile watershed at 8.7 ka in our record. This was characterized by a switch in sediment provenance towards higher contributions by the White Nile and Saharan areas [13] (Fig. 3b) and by a retreat of the savannah with lowest grass content attained between 7 and 6 ka BP (Fig. 3a). Human populations were thus probably forced to adapt to the rapid degradation of the environment by relying increasingly on domesticated animals and by migrating to areas allowing sustainable herding, which supports the hypothesis that human-environment relationships played a significant role in the domestication process [4], [55].

The first evidence of domesticated cattle in NE Africa lags behind the Fertile Crescent occurrence by ∼2000 years [8]. It thus seems that early Neolithic populations in the Fertile Crescent benefited from the favorable conditions during the AHP to implement agriculture and then herding while NE African populations thrived in the Green Sahara and only turned to pastoralism when the living conditions became more difficult [6], [8].

Around 6 ka BP, widespread ritual burial of cattle, known as the ‘African Cattle Complex’ [55], and the first evidence of dairying in the Central Sahara [56] demonstrated that pastoralism had spread in the Sahara and had become a well-established subsistence method. At this time, the savannah had almost entirely disappeared from the Sahara, which became increasingly arid between 6 and 2.5 ka BP, as shown by the radiogenic strontium signature of detrital sediments (Fig. 3b), as well as grain-size data from core P362/2-33 [13]. From 5 ka BP onwards, the population in the Nile Valley increased gradually and gave rise to the Egyptian Pharaonic Kingdoms and to the subsequent development of agriculture [57]. In our record, this period is marked by the most arid conditions as evidenced by the highest amounts of eolian dust found in the sediments between ∼5 and 3 ka BP (Fig. 3b and ref. [13]).

A pronounced shift in sediment provenance towards a Blue Nile source occurred at 2.5 ka BP (Fig. 3a) and reflects the re-establishment of seasonal rainfalls in the Blue Nile drainage basin after 3 ka BP due to an increase in autumn insolation [13], [46], [57]. However, these floods were much less vigorous than those during the Early Holocene (as documented by the lower sedimentation rates, Fig. 2f), because summer insolation that mainly controls monsoon strength at the Blue Nile source was lower during the Late Holocene than during the Early Holocene [13].

Our new continuous high-resolution record of changes in vegetation, erosion and river runoff within the Nile River drainage area allows to place the main steps of Neolithic human evolution into a detailed environmental context. An abrupt degradation of the vegetation and a switch in sediment source during the Early Holocene (at 8.7 ka BP) occurred without a significant decrease in river runoff and precipitation. Our record therefore confirms the large regional heterogeneity of environmental change in NE Africa and provides new insights into the climatic mechanisms involved in the termination of the North African Humid Period, in particular the existence of threshold mechanisms. The degradation of the vegetation probably had a considerable impact on the initiation of cattle domestication in North Africa and confirms the hypothesis that aridification in North Africa had a profound impact on human evolution.

## Supporting Information

S1 Fig **Reconstruction of δ^18^O of surface seawater**. A: δ^18^O measured on the planktonic foraminifera *Globigerinoides ruber*. B: Surface seawater temperatures (SST) reconstructed using the alkenone insaturation index (Uk_37_′). Measured points are indicated by the filled diamonds; values in between measured points were estimated using linear interpolation provided by the Analyseries software package (http://www.lsce.ipsl.fr/Phocea/Page/index.php?id=3) in order to provide the same spatial resolution as the δ^18^O record. C: δ^18^O of the surface seawater (δ^18^O_SW_), as compared to the present-day values for the Nile freshwater and the eastern Mediterranean surface seawater (0–200 m water depth) [33], [36].
(TIFF)

S2 Fig **Isotopic composition of long-chain odd** ***n-*** **alkanes**. A: Carbon isotope composition (δ^13^C) of *n-*C_27_; B: δ^13^C of *n-*C_29_; C: δ^13^C of *n-*C_31_ and D: δ^13^C of *n-*C_33_. E: Weighted average of the δ^13^C of *n-*C_27_-*n-*C_33_.
(TIFF)

S3 Fig **Content and accumulation rates (AR) of lipid biomarkers.** A: AR (dashed orange line) and concentration (thick red line) of long-chain odd n-alkanes. B: AR (dashed green line) and concentration (thick green line) of branched GDGTs, with the structure of the dominant branched GDGTs. C: AR (dashed blue line) and concentration (thick blue line) of crenarchaeol, with its structure. D: BIT index.
(TIFF)

S1 Table **Surface seawater properties**. Stable oxygen isotopes (δ^18^O) for the planktonic foraminifera *Globigerinoides ruber*; surface seawater temperature (SST) as evaluated using the alkenone paleothermometer (Uk_37_′) for 39 samples and linearly interpolated; δ^18^O of the surface seawater (δ^18^O_SW_) obtained using the paleotemperature equation of ref. [31].
(DOC)

S2 Table **Radiogenic Nd and Sr isotopes for the total dissolutions.** The radiogenic Nd and Sr isotopes of the total dissolutions is the signal carried by the siliciclastic (detrital) fraction of the sediments. All Nd and Sr isotope ratios are given with a 2σ external reproducibility.
(DOC)

S3 Table **Carbon isotope ratios in long-chain odd** ***n-*** **alkanes**. The weighted average of the δ^13^C of the *n-*C_27_, *n-*C_29_, *n-*C_31_ and *n-*C_33_ *n-*alkanes is given with the standard deviation. The percentage of C_4_ plants was calculated using the mixing model by ref. [24].
(DOC)

S4 Table **Concentration and accumulation rates of GDGTs and BIT index.**
(DOC)
