# Supplementary figures and images for: Asynchronous Changes in Vegetation, Runoff and Erosion in the Nile River Watershed during the Holocene
Source: PLoS One. 2014 Dec 31;9(12):e115958. doi: 10.1371/journal.pone.0115958 (PMC4281134; doi:10.1371/journal.pone.0115958)

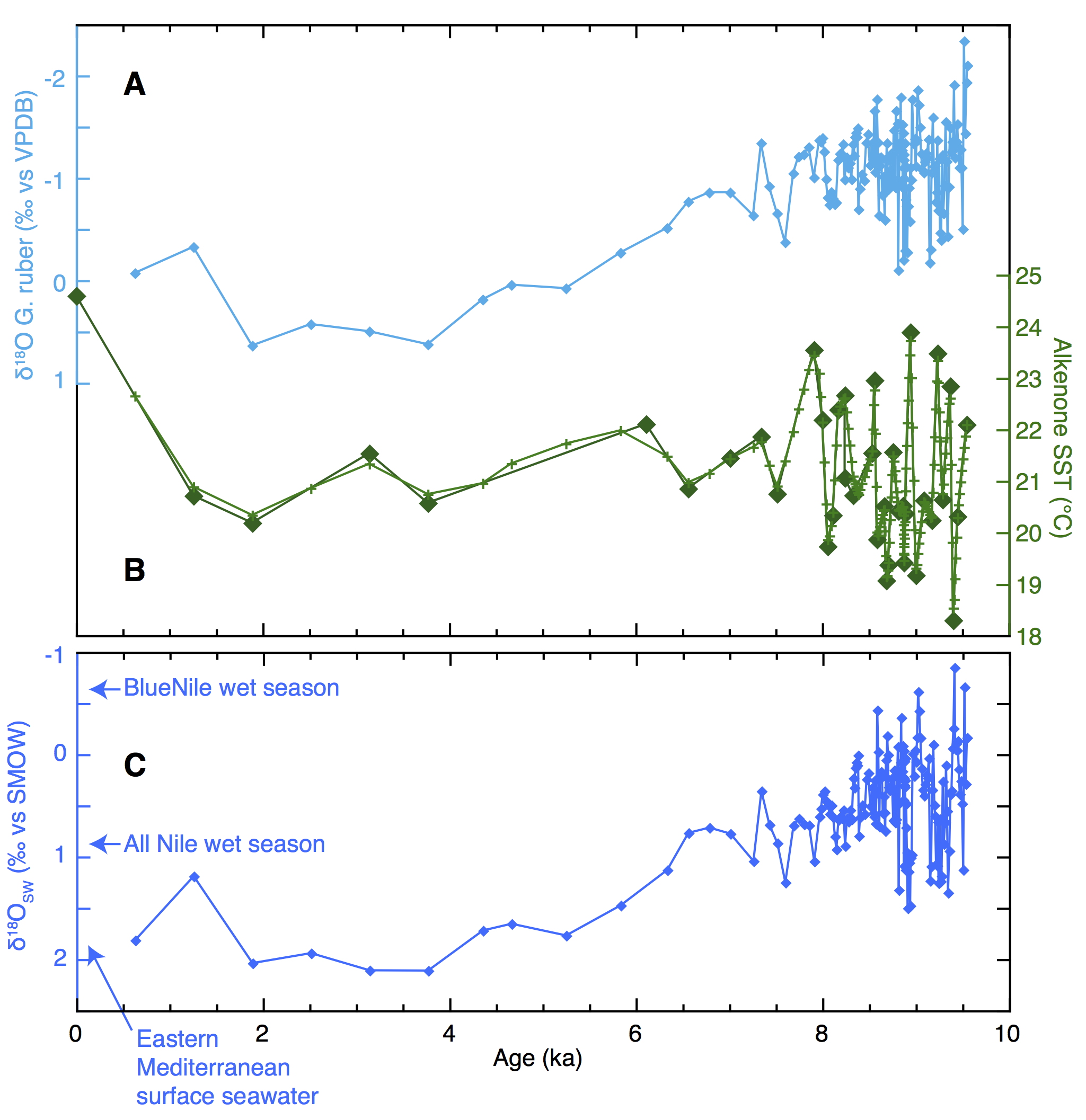

Supplement: S1 Fig — Reconstruction of δ18O of surface seawater. A: δ18O measured on the planktonic foraminifera Globigerinoides ruber. B: Surface seawater temperatures (SST) reconstructed using the alkenone insaturation index (Uk37′). Measured points are indicated by the filled diamonds; values in between measured points were estimated using linear interpolation provided by the Analyseries software package (http://www.lsce.ipsl.fr/Phocea/Page/index.php?id=3) in order to provide the same spatial resolution as the δ18O record. C: δ18O of the surface seawater (δ18OSW), as compared to the present-day values for the Nile freshwater and the eastern Mediterranean surface seawater (0–200 m water depth) [33], [36]. (TIFF) [file pone.0115958.s001.tiff]

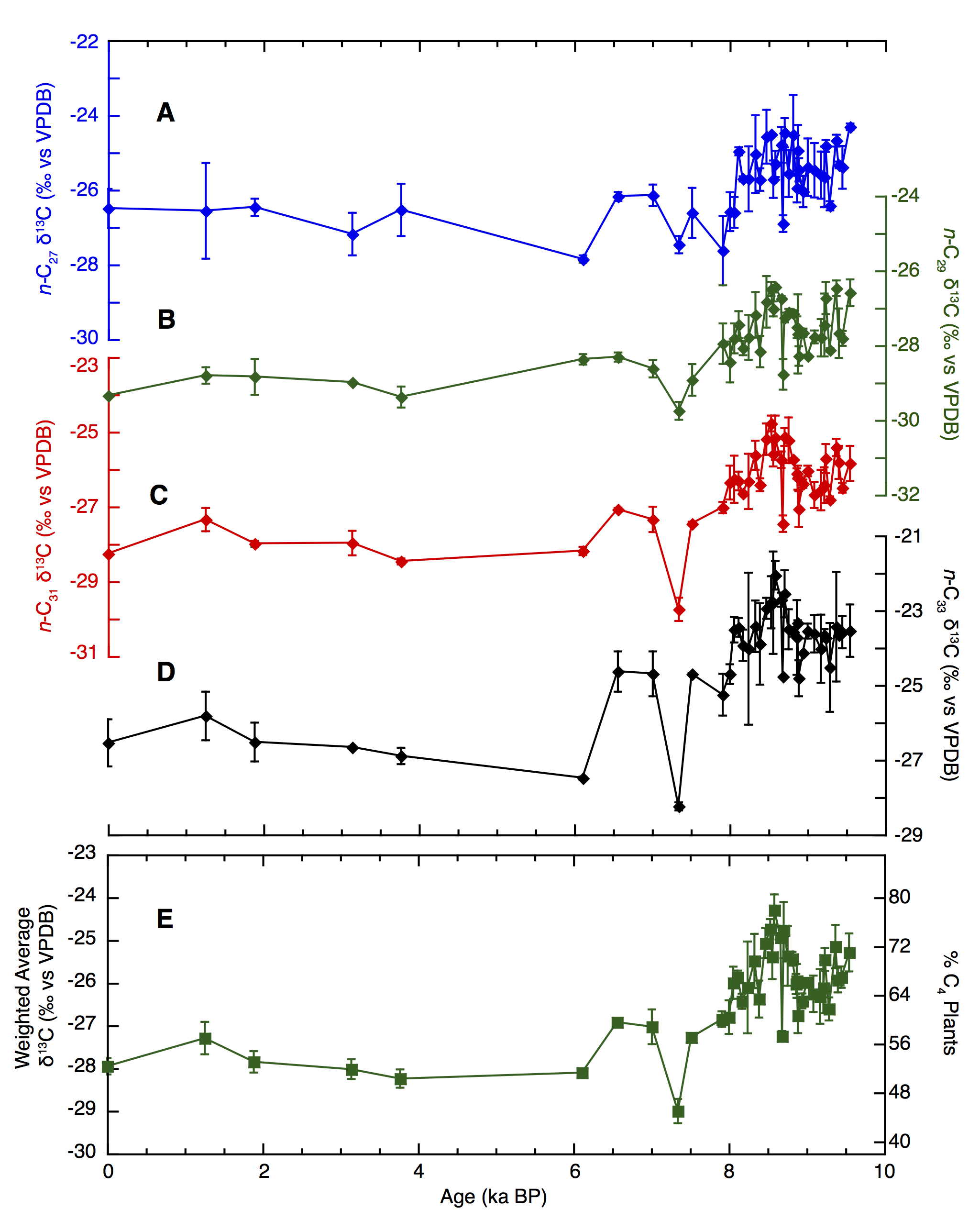

Supplement: S2 Fig — Isotopic composition of long-chain odd n- alkanes. A: Carbon isotope composition (δ13C) of n-C27; B: δ13C of n-C29; C: δ13C of n-C31 and D: δ13C of n-C33. E: Weighted average of the δ13C of n-C27-n-C33. (TIFF) [file pone.0115958.s002.tiff]

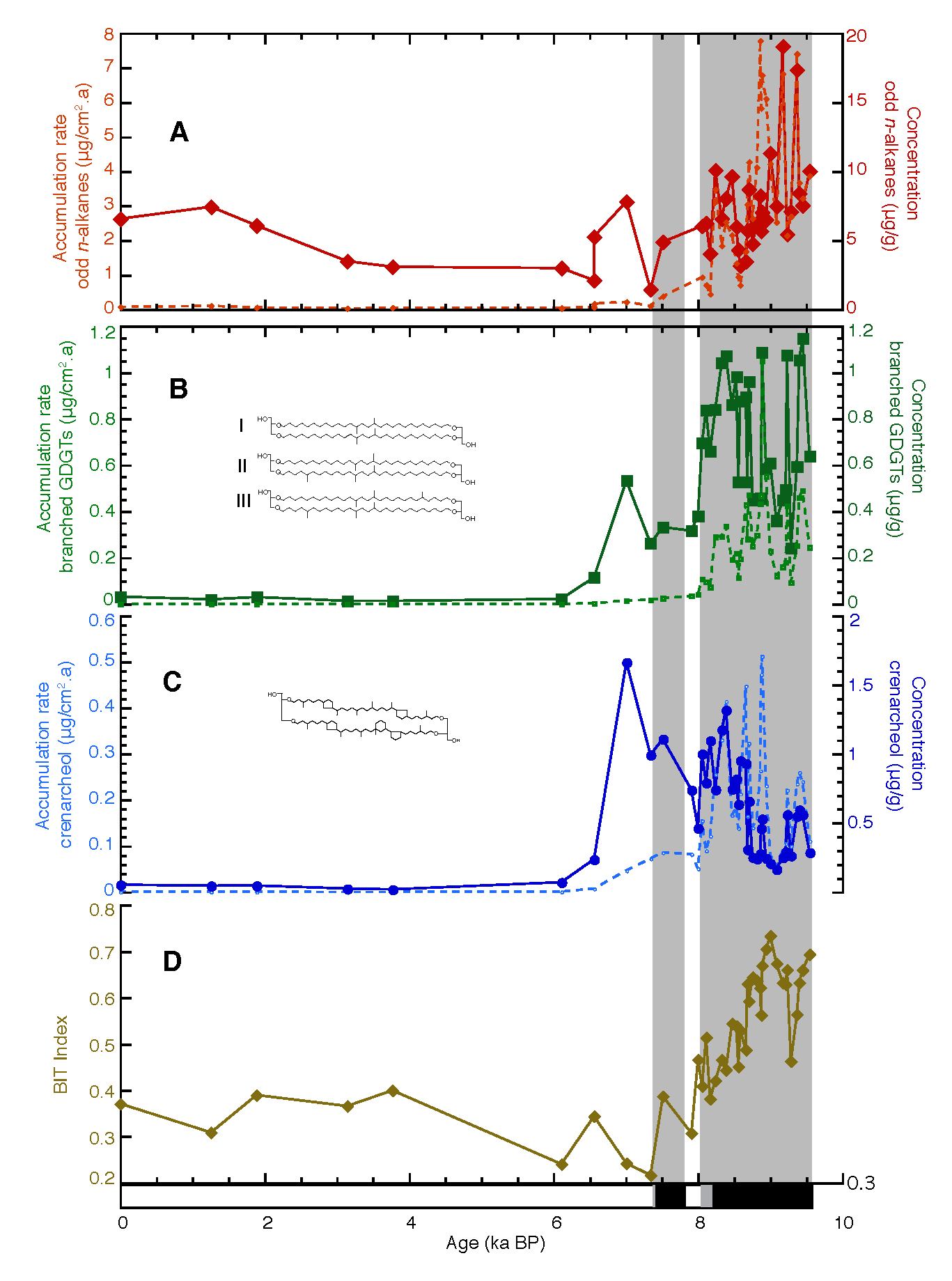

Supplement: S3 Fig — Content and accumulation rates (AR) of lipid biomarkers. A: AR (dashed orange line) and concentration (thick red line) of long-chain odd n-alkanes. B: AR (dashed green line) and concentration (thick green line) of branched GDGTs, with the structure of the dominant branched GDGTs. C: AR (dashed blue line) and concentration (thick blue line) of crenarchaeol, with its structure. D: BIT index. (TIFF) [file pone.0115958.s003.tiff]
