# Supplementary material for: Asynchronous Changes in Vegetation, Runoff and Erosion in the Nile River Watershed during the Holocene
Source: PLoS One. 2014 Dec 31;9(12):e115958. doi: 10.1371/journal.pone.0115958 (PMC4281134; doi:10.1371/journal.pone.0115958)
Supplement: S1 Table — Surface seawater properties. Stable oxygen isotopes (δ18O) for the planktonic foraminifera Globigerinoides ruber; surface seawater temperature (SST) as evaluated using the alkenone paleothermometer (Uk37′) for 39 samples and linearly interpolated; δ18O of the surface seawater (δ18OSW) obtained using the paleotemperature equation of ref. [31]. (DOC) [file pone.0115958.s004.doc]

| Depth (cm) | Age (ka) | δ^18^O (‰ vs V-PDB) | Uk_37_’ | SST (°C) | SST (°C) (interpolated) | δ^18^O_SW_ (‰ vs SMOW) |
| --- | --- | --- | --- | --- | --- | --- |
|  |  |  | 0.86 | 24.60 |  |  |
| 5 | 0.63 | -0.07 |  |  | 22.66 | 1.81 |
| 10 | 1.26 | -0.33 | 0.69 | 20.72 | 20.89 | 1.19 |
| 15 | 1.88 | 0.63 | 0.67 | 20.19 | 20.34 | 2.04 |
| 20 | 2.51 | 0.43 |  |  | 20.87 | 1.94 |
| 25 | 3.14 | 0.50 | 0.73 | 21.54 | 21.34 | 2.11 |
| 30 | 3.77 | 0.62 | 0.69 | 20.58 | 20.75 | 2.11 |
| 35 | 4.36 | 0.19 |  |  | 20.96 | 1.72 |
| 40 | 4.66 | 0.04 |  |  | 21.35 | 1.65 |
| 45 | 5.25 | 0.08 |  |  | 21.73 | 1.77 |
| 50 | 5.84 | -0.27 | 0.76 | 22.11 | 21.99 | 1.47 |
| 55 | 6.34 | -0.51 |  |  | 21.49 | 1.13 |
| 60 | 6.56 | -0.77 | 0.70 | 20.86 | 20.97 | 0.77 |
| 65 | 6.79 | -0.86 |  |  | 21.15 | 0.71 |
| 70 | 7.01 | -0.86 | 0.73 | 21.45 | 21.44 | 0.77 |
| 75 | 7.26 | -0.64 |  |  | 21.66 | 1.04 |
| 80 | 7.34 | -1.34 | 0.75 | 21.87 | 21.77 | 0.36 |
| 85 | 7.43 | -0.92 |  |  | 21.31 | 0.68 |
| 90 | 7.51 | -0.66 | 0.70 | 20.76 | 20.91 | 0.87 |
| 95 | 7.60 | -0.37 |  |  | 21.39 | 1.25 |
| 100 | 7.69 | -1.05 |  |  | 21.96 | 0.69 |
| 103 | 7.75 | -1.21 |  |  | 22.41 | 0.62 |
| 106 | 7.80 | -1.23 |  |  | 22.79 | 0.68 |
| 109 | 7.85 | -1.30 |  |  | 23.17 | 0.69 |
| 112 | 7.91 | -1.01 | 0.82 | 23.55 | 23.45 | 1.04 |
| 115 | 7.96 | -1.37 |  |  | 23.10 | 0.61 |
| 118 | 7.98 | -1.36 |  |  | 22.65 | 0.53 |
| 121 | 8.00 | -1.39 | 0.76 | 22.20 | 22.15 | 0.39 |
| 124 | 8.02 | -1.26 |  |  | 21.38 | 0.36 |
| 127 | 8.04 | -0.99 |  |  | 20.56 | 0.46 |
| 130 | 8.06 | -0.81 | 0.65 | 19.74 | 19.87 | 0.49 |
| 133 | 8.08 | -0.74 |  |  | 19.94 | 0.58 |
| 136 | 8.09 | -0.87 |  |  | 20.14 | 0.50 |
| 139 | 8.11 | -0.80 | 0.68 | 20.34 | 20.40 | 0.61 |
| 142 | 8.13 | -0.75 |  |  | 21.02 | 0.80 |
| 145 | 8.15 | -0.76 |  |  | 21.71 | 0.93 |
| 148 | 8.17 | -1.18 | 0.77 | 22.39 | 22.32 | 0.64 |
| 151 | 8.19 | -1.24 |  |  | 22.46 | 0.61 |
| 154 | 8.20 | -1.24 |  |  | 22.53 | 0.62 |
| 157 | 8.22 | -1.33 |  |  | 22.60 | 0.54 |
| 160 | 8.24 | -0.99 | 0.78 | 22.67 | 22.62 | 0.89 |
| 163 | 8.26 | -1.20 |  |  | 22.35 | 0.62 |
| 166 | 8.28 | -1.10 |  |  | 22.03 | 0.65 |
| 169 | 8.29 | -1.15 |  |  | 21.71 | 0.54 |
| 172 | 8.31 | -0.99 |  |  | 21.39 | 0.63 |
| 175 | 8.33 | -1.33 | 0.71 | 21.07 | 21.10 | 0.23 |
| 178 | 8.34 | -1.22 |  |  | 21.01 | 0.32 |
| 181 | 8.35 | -1.40 |  |  | 20.95 | 0.13 |
| 184 | 8.36 | -1.45 |  |  | 20.90 | 0.07 |
| 187 | 8.37 | -1.40 |  |  | 20.84 | 0.11 |
| Depth (cm) | Age (ka) | δ^18^O (‰ vs V-PDB) | Uk_37_’ | SST (°C) | SST (°C) (interpolated) | δ^18^O_SW_ (‰ vs SMOW) |
| 190 | 8.38 | -1.49 |  |  | 20.78 | 0.01 |
| 193 | 8.39 | -0.69 | 0.69 | 20.73 | 20.75 | 0.79 |
| 196 | 8.40 | -0.90 |  |  | 20.84 | 0.61 |
| 199 | 8.42 | -1.04 |  |  | 20.96 | 0.49 |
| 203 | 8.45 | -0.98 |  |  | 21.09 | 0.58 |
| 206 | 8.47 | -1.35 |  |  | 21.21 | 0.24 |
| 209 | 8.49 | -1.43 |  |  | 21.32 | 0.18 |
| 212 | 8.51 | -1.13 |  |  | 21.44 | 0.50 |
| 215 | 8.53 | -1.34 | 0.73 | 21.55 | 21.59 | 0.32 |
| 218 | 8.54 | -1.19 |  |  | 22.02 | 0.57 |
| 221 | 8.55 | -1.25 |  |  | 22.49 | 0.61 |
| 224 | 8.56 | -1.66 | 0.79 | 22.96 | 22.77 | 0.25 |
| 227 | 8.57 | -1.06 |  |  | 21.93 | 0.67 |
| 230 | 8.58 | -1.15 |  |  | 20.90 | 0.37 |
| 233 | 8.59 | -1.77 | 0.66 | 19.87 | 20.01 | -0.43 |
| 236 | 8.60 | -1.35 |  |  | 19.95 | -0.03 |
| 239 | 8.61 | -0.64 |  |  | 20.04 | 0.70 |
| 242 | 8.62 | -1.12 |  |  | 20.12 | 0.23 |
| 245 | 8.63 | -1.21 |  |  | 20.20 | 0.17 |
| 248 | 8.64 | -1.19 |  |  | 20.28 | 0.20 |
| 251 | 8.65 | -0.84 |  |  | 20.36 | 0.57 |
| 254 | 8.66 | -1.02 |  |  | 20.44 | 0.41 |
| 257 | 8.66 | -0.86 | 0.69 | 20.52 | 20.45 | 0.57 |
| 260 | 8.67 | -0.59 |  |  | 20.04 | 0.75 |
| 263 | 8.68 | -1.19 |  |  | 19.55 | 0.05 |
| 266 | 8.69 | -0.93 | 0.62 | 19.07 | 19.15 | 0.23 |
| 269 | 8.69 | -1.34 |  |  | 19.17 | -0.18 |
| 272 | 8.70 | -1.18 |  |  | 19.28 | 0.00 |
| 275 | 8.71 | -0.89 | 0.63 | 19.38 | 19.42 | 0.32 |
| 278 | 8.72 | -0.95 |  |  | 19.82 | 0.35 |
| 281 | 8.73 | -1.08 |  |  | 20.26 | 0.31 |
| 284 | 8.74 | -1.24 |  |  | 20.69 | 0.23 |
| 287 | 8.75 | -1.24 |  |  | 21.13 | 0.33 |
| 290 | 8.76 | -1.00 | 0.73 | 21.57 | 21.49 | 0.64 |
| 293 | 8.77 | -1.47 |  |  | 21.39 | 0.15 |
| 296 | 8.78 | -0.92 |  |  | 21.21 | 0.66 |
| 299 | 8.79 | -0.91 |  |  | 21.01 | 0.63 |
| 301 | 8.79 | -1.66 |  |  |  |  |
| 303 | 8.80 | -1.29 |  |  | 20.80 | 0.21 |
| 306 | 8.81 | -1.54 |  |  | 20.60 | -0.08 |
| 309 | 8.81 | -0.10 | 0.68 | 20.42 | 20.45 | 1.32 |
| 312 | 8.82 | -0.96 |  |  | 20.44 | 0.47 |
| 315 | 8.83 | -1.28 |  |  | 20.45 | 0.15 |
| 318 | 8.84 | -1.35 |  |  | 20.46 | 0.07 |
| 321 | 8.84 | -1.79 |  |  | 20.47 | -0.36 |
| 324 | 8.85 | -1.20 |  |  | 20.49 | 0.23 |
| 327 | 8.85 | -1.27 |  |  | 20.50 | 0.16 |
| 330 | 8.86 | -1.53 |  |  | 20.51 | -0.09 |
| 333 | 8.86 | -1.00 | 0.69 | 20.53 | 20.50 | 0.44 |
| 336 | 8.87 | -1.44 |  |  | 20.34 | -0.04 |
| Depth (cm) | Age (ka) | δ^18^O (‰ vs V-PDB) | Uk_37_’ | SST (°C) | SST (°C) (interpolated) | δ^18^O_SW_ (‰ vs SMOW) |
| 339 | 8.87 | -0.90 |  |  | 20.16 | 0.46 |
| 342 | 8.87 | -1.10 |  |  | 19.97 | 0.23 |
| 345 | 8.87 | -0.20 |  |  | 19.79 | 1.09 |
| 348 | 8.87 | -1.00 |  |  | 19.60 | 0.25 |
| 351 | 8.87 | -0.92 | 0.63 | 19.42 | 19.46 | 0.30 |
| 354 | 8.87 | -1.18 |  |  | 19.58 | 0.07 |
| 357 | 8.88 | -1.23 |  |  | 19.74 | 0.05 |
| 360 | 8.88 | -1.00 |  |  | 19.90 | 0.32 |
| 363 | 8.88 | -1.09 |  |  | 20.06 | 0.25 |
| 366 | 8.89 | -1.34 | 0.68 | 20.38 | 20.22 | 0.04 |
| 369 | 8.89 | -0.29 |  |  | 20.41 | 1.12 |
| 372 | 8.89 | -0.79 |  |  | 20.82 | 0.71 |
| 375 | 8.89 | -1.12 |  |  | 21.26 | 0.48 |
| 378 | 8.90 | -0.73 |  |  | 21.70 | 0.96 |
| 381 | 8.91 | -0.28 |  |  | 22.14 | 1.50 |
| 384 | 8.92 | -0.73 |  |  | 22.58 | 1.14 |
| 387 | 8.93 | -0.91 |  |  | 23.01 | 1.05 |
| 390 | 8.94 | -0.58 |  |  | 23.45 | 1.48 |
| 393 | 8.94 | -1.10 | 0.83 | 23.89 | 23.73 | 1.01 |
| 396 | 8.95 | -0.98 |  |  | 23.01 | 0.98 |
| 399 | 8.96 | -1.77 |  |  | 22.05 | -0.01 |
| 403 | 8.98 | -1.34 | 0.62 | 19.18 | 21.02 | 0.21 |
| 406 | 8.99 | -1.39 |  |  | 20.06 | -0.04 |
| 409 | 9.00 | -1.12 |  |  | 19.31 | 0.07 |
| 412 | 9.01 | -1.37 |  |  | 19.39 | -0.17 |
| 415 | 9.02 | -1.86 |  |  | 19.59 | -0.61 |
| 418 | 9.04 | -1.72 |  |  | 19.80 | -0.43 |
| 421 | 9.05 | -1.50 |  |  | 20.01 | -0.16 |
| 424 | 9.06 | -1.24 |  |  | 20.22 | 0.14 |
| 427 | 9.07 | -1.08 |  |  | 20.42 | 0.34 |
| 430 | 9.09 | -1.06 | 0.69 | 20.63 | 20.60 | 0.40 |
| 433 | 9.10 | -1.16 |  |  | 20.58 | 0.29 |
| 436 | 9.11 | -1.25 |  |  | 20.52 | 0.20 |
| 439 | 9.13 | -1.20 |  |  | 20.47 | 0.23 |
| 442 | 9.14 | -1.38 |  |  | 20.41 | 0.04 |
| 445 | 9.15 | -0.18 |  |  | 20.36 | 1.23 |
| 448 | 9.16 | -0.30 |  |  | 20.30 | 1.09 |
| 451 | 9.18 | -1.06 | 0.67 | 20.25 | 20.32 | 0.34 |
| 454 | 9.19 | -1.59 |  |  | 20.79 | -0.10 |
| 457 | 9.20 | -1.12 |  |  | 21.33 | 0.49 |
| 460 | 9.21 | -1.12 |  |  | 21.87 | 0.60 |
| 463 | 9.22 | -0.77 |  |  | 22.41 | 1.07 |
| 466 | 9.23 | -0.86 |  |  | 22.95 | 1.08 |
| 469 | 9.23 | -1.37 | 0.81 | 23.49 | 23.35 | 0.66 |
| 472 | 9.24 | -0.69 |  |  | 22.92 | 1.25 |
| 475 | 9.25 | -1.19 |  |  | 22.35 | 0.63 |
| 478 | 9.26 | -0.47 |  |  | 21.78 | 1.24 |
| 481 | 9.28 | -0.40 |  |  | 21.21 | 1.19 |
| 484 | 9.29 | -1.23 | 0.69 | 20.65 | 20.75 | 0.26 |
| 487 | 9.30 | -0.66 |  |  | 20.95 | 0.87 |
| Depth (cm) | Age (ka) | δ^18^O (‰ vs V-PDB) | Uk_37_’ | SST (°C) | SST (°C) (interpolated) | δ^18^O_SW_ (‰ vs SMOW) |
| 490 | 9.31 | -0.93 |  |  | 21.25 | 0.66 |
| 493 | 9.32 | -1.55 |  |  | 21.54 | 0.11 |
| 496 | 9.33 | -1.17 |  |  | 21.84 | 0.55 |
| 499 | 9.34 | -0.43 |  |  | 22.17 | 1.35 |
| 503 | 9.36 | -0.92 |  |  | 22.52 | 0.94 |
| 506 | 9.37 | -1.49 | 0.79 | 22.84 | 22.62 | 0.39 |
| 509 | 9.38 | -1.26 |  |  | 21.33 | 0.35 |
| 512 | 9.39 | -1.36 |  |  | 19.82 | -0.06 |
| 515 | 9.40 | -1.28 | 0.58 | 18.30 | 18.54 | -0.25 |
| 518 | 9.41 | -1.91 |  |  | 18.70 | -0.85 |
| 521 | 9.42 | -1.20 |  |  | 19.11 | -0.05 |
| 524 | 9.43 | -1.34 |  |  | 19.51 | -0.11 |
| 527 | 9.44 | -1.36 |  |  | 19.92 | -0.04 |
| 530 | 9.45 | -1.53 | 0.68 | 20.32 | 20.30 | -0.13 |
| 533 | 9.46 | -1.30 |  |  | 20.54 | 0.14 |
| 536 | 9.47 | -1.10 |  |  | 20.76 | 0.39 |
| 539 | 9.48 | -1.28 |  |  | 20.99 | 0.26 |
| 542 | 9.49 | -1.11 |  |  | 21.21 | 0.48 |
| 545 | 9.51 | -0.50 |  |  | 21.43 | 1.13 |
| 548 | 9.52 | -2.34 |  |  | 21.66 | -0.66 |
| 551 | 9.53 | -1.44 |  |  | 21.88 | 0.29 |
| 554 | 9.55 | -1.94 | 0.76 | 22.10 | 22.10 | -0.17 |
| 557 | 9.56 | -2.10 |  |  |  |  |
