# Supplementary material for: Asynchronous Changes in Vegetation, Runoff and Erosion in the Nile River Watershed during the Holocene
Source: PLoS One. 2014 Dec 31;9(12):e115958. doi: 10.1371/journal.pone.0115958 (PMC4281134; doi:10.1371/journal.pone.0115958)
Supplement: S2 Table — Radiogenic Nd and Sr isotopes for the total dissolutions. The radiogenic Nd and Sr isotopes of the total dissolutions is the signal carried by the siliciclastic (detrital) fraction of the sediments. All Nd and Sr isotope ratios are given with a 2σ external reproducibility. (DOC) [file pone.0115958.s005.doc]

| Depth (cm) | Age (ka) | ^143^Nd/^144^Nd TD | εNd TD | 2σ | ^87^Sr/^86^Sr TD | 2σ |
| --- | --- | --- | --- | --- | --- | --- |
| 0 | 0.00 | 0.512346 | -5.69 | 0.23 | 0.710127 | 0.000024 |
| 5 | 0.63 | 0.512606 | -0.62 | 0.23 | 0.707161 | 0.000027 |
| 10 | 1.26 | 0.512557 | -1.57 | 0.39 | 0.709448 | 0.000048 |
| 20 | 2.51 | 0.512355 | -5.53 | 0.52 | 0.712386 | 0.000039 |
| 30 | 3.77 | 0.512335 | -5.91 | 0.16 |  |  |
| 40 | 4.94 | 0.512352 | -5.57 | 0.52 | 0.710798 | 0.000016 |
| 45 | 5.53 | 0.512417 | -4.30 | 0.52 | 0.710042 | 0.000012 |
| 50 | 6.11 | 0.512446 | -3.75 | 0.39 | 0.710599 | 0.000048 |
| 55 | 6.34 | 0.512376 | -5.11 | 0.52 | 0.709993 | 0.000027 |
| 60 | 6.56 | 0.512359 | -5.44 | 0.52 | 0.709477 | 0.000009 |
| 65 | 6.79 | 0.512412 | -4.40 | 0.52 |  |  |
| 70 | 7.01 | 0.512435 | -3.96 | 0.39 | 0.709967 | 0.000048 |
| 75 | 7.24 | 0.512455 | -3.58 | 0.20 | 0.709926 | 0.000033 |
| 85 | 7.43 | 0.512433 | -4.01 | 0.39 | 0.709954 | 0.000048 |
| 118 | 7.98 | 0.512427 | -4.11 | 0.20 | 0.709932 | 0.000033 |
| 151 | 8.19 | 0.512444 | -3.79 | 0.20 | 0.708638 | 0.000033 |
| 181 | 8.35 | 0.512445 | -3.77 | 0.20 | 0.708942 | 0.000033 |
| 221 | 8.55 | 0.512421 | -4.23 | 0.20 | 0.710062 | 0.000030 |
| 263 | 8.68 | 0.512470 | -3.28 | 0.33 | 0.707306 | 0.000033 |
| 293 | 8.77 | 0.512494 | -2.80 | 0.20 | 0.707230 | 0.000033 |
| 348 | 8.87 | 0.512486 | -2.96 | 0.20 | 0.708130 | 0.000033 |
| 396 | 8.95 |  |  |  | 0.707072 | 0.000026 |
| 424 | 9.06 | 0.512465 | -3.37 | 0.20 | 0.707073 | 0.000033 |
| 457 | 9.21 | 0.512492 | -2.84 | 0.20 | 0.708251 | 0.000033 |
| 503 | 9.36 |  |  |  | 0.708070 | 0.000033 |
| 551 | 9.53 | 0.512442 | -3.82 | 0.20 | 0.707553 | 0.000033 |
