# Supplementary material for: Asynchronous Changes in Vegetation, Runoff and Erosion in the Nile River Watershed during the Holocene
Source: PLoS One. 2014 Dec 31;9(12):e115958. doi: 10.1371/journal.pone.0115958 (PMC4281134; doi:10.1371/journal.pone.0115958)
Supplement: S3 Table — Carbon isotope ratios in long-chain odd n- alkanes. The weighted average of the δ13C of the n-C27, n-C29, n-C31 and n-C33 n-alkanes is given with the standard deviation. The percentage of C4 plants was calculated using the mixing model by ref. [24]. (DOC) [file pone.0115958.s006.doc]

| Depth | Age (ka) | Weighted Average δ^13^C_wax_ | Standard deviation | %C_4_ Plants |
| --- | --- | --- | --- | --- |
| 0 | 0.00 | -27.95 | 0.19 | 52 |
| 10 | 1.26 | -27.29 | 0.38 | 57 |
| 15 | 1.88 | -27.84 | 0.25 | 53 |
| 25 | 3.14 | -28.02 | 0.23 | 52 |
| 30 | 3.77 | -28.24 | 0.21 | 50 |
| 50 | 6.11 | -28.10 | 0.00 | 51 |
| 60 | 6.56 | -26.91 | 0.00 | 60 |
| 70 | 7.01 | -27.02 | 0.41 | 59 |
| 80 | 7.34 | -29.00 | 0.28 | 44 |
| 90 | 7.51 | -27.27 | 0.09 | 57 |
| 112 | 7.91 | -26.85 | 0.18 | 60 |
| 121 | 8.00 | -26.80 | 0.39 | 61 |
| 130 | 8.06 | -26.00 | 0.38 | 67 |
| 139 | 8.11 | -25.86 | 0.15 | 68 |
| 148 | 8.17 | -26.43 | 0.18 | 64 |
| 160 | 8.24 | -26.11 | 1.07 | 66 |
| 175 | 8.33 | -25.48 | 0.63 | 71 |
| 193 | 8.39 | -26.38 | 0.43 | 64 |
| 206 | 8.47 | -25.07 | 0.35 | 74 |
| 215 | 8.53 | -24.74 | 0.24 | 76 |
| 224 | 8.56 | -25.39 | 0.52 | 71 |
| 233 | 8.59 | -24.29 | 0.37 | 80 |
| 257 | 8.66 | -24.93 | 0.06 | 75 |
| 266 | 8.69 | -27.24 | 0.12 | 58 |
| 275 | 8.71 | -24.76 | 0.66 | 76 |
| 290 | 8.76 | -25.37 | 0.70 | 72 |
| 309 | 8.81 | -25.43 | 0.16 | 71 |
| 333 | 8.86 | -26.02 | 0.24 | 67 |
| 351 | 8.87 | -25.95 | 0.39 | 67 |
| 369 | 8.89 | -26.76 | 0.41 | 61 |
| 393 | 8.94 | -26.42 | 0.17 | 64 |
| 409 | 9.00 | -25.98 | 0.09 | 67 |
| 430 | 9.09 | -26.25 | 0.42 | 65 |
| 451 | 9.18 | -26.32 | 0.64 | 64 |
| 463 | 9.22 | -26.11 | 0.59 | 66 |
| 469 | 9.23 | -25.45 | 0.27 | 71 |
| 484 | 9.29 | -26.61 | 0.27 | 62 |
| 506 | 9.37 | -25.15 | 0.51 | 73 |
| 515 | 9.40 | -25.93 | 0.29 | 67 |
| 530 | 9.45 | -25.86 | 0.25 | 68 |
| 554 | 9.55 | -25.29 | 0.45 | 72 |
