# Supplementary material for: Asynchronous Changes in Vegetation, Runoff and Erosion in the Nile River Watershed during the Holocene
Source: PLoS One. 2014 Dec 31;9(12):e115958. doi: 10.1371/journal.pone.0115958 (PMC4281134; doi:10.1371/journal.pone.0115958)
Supplement: S4 Table — Concentration and accumulation rates of GDGTs and BIT index. (DOC) [file pone.0115958.s007.doc]

| Depth (cm) | Age (ka) | AR GDGT-0 (ng/cm^2^.a) | Conc. Crenarch. (ng/g) | AR Crenarch. (ng/cm^2^.a) | Conc. Branch. GDGTs (ng/g) | AR Branch. GDGTs (ng/cm^2^.a) | BIT | std dev. |
| --- | --- | --- | --- | --- | --- | --- | --- | --- |
| 0 | 0.00 | 0.24 | 53.32 | 0.60 | 31.62 | 0.36 | 0.37 | 0.01 |
| 10 | 1.26 | 0.25 | 46.16 | 0.69 | 20.36 | 0.30 | 0.31 | 0.00 |
| 15 | 1.88 | 0.17 | 47.29 | 0.45 | 30.40 | 0.29 | 0.39 | 0.01 |
| 25 | 3.14 | 0.10 | 25.85 | 0.29 | 13.66 | 0.16 | 0.37 | 0.02 |
| 30 | 3.77 | 0.14 | 20.13 | 0.36 | 13.42 | 0.24 | 0.40 | 0.02 |
| 50 | 6.11 | 0.40 | 72.07 | 1.03 | 22.63 | 0.32 | 0.24 | 0.01 |
| 60 | 6.56 | 3.19 | 237.64 | 7.74 | 114.86 | 3.74 | 0.34 | 0.00 |
| 70 | 7.01 | 17.66 | 1665.61 | 47.05 | 533.11 | 15.06 | 0.24 | 0.01 |
| 80 | 7.34 | 33.37 | 995.19 | 73.18 | 262.79 | 19.32 | 0.22 | 0.01 |
| 90 | 7.51 | 48.47 | 1110.26 | 86.64 | 332.62 | 25.96 | 0.39 | 0.00 |
| 112 | 7.91 | 30.67 | 740.37 | 82.58 | 317.23 | 35.38 | 0.31 | 0.00 |
| 121 | 8.00 | 23.36 | 463.86 | 51.40 | 382.25 | 42.35 | 0.47 | 0.02 |
| 130 | 8.06 | 75.33 | 1001.32 | 154.88 | 696.36 | 107.71 | 0.41 | 0.01 |
| 139 | 8.11 | 45.74 | 793.62 | 90.50 | 837.47 | 95.50 | 0.51 | 0.00 |
| 148 | 8.17 | 54.93 | 1098.93 | 122.11 | 660.97 | 73.45 | 0.38 | 0.01 |
| 160 | 8.24 | 119.16 | 743.10 | 256.23 | 841.96 | 290.31 | 0.42 | 0.01 |
| 175 | 8.33 | 148.40 | 1176.99 | 331.04 | 1042.51 | 293.22 | 0.47 | 0.00 |
| 193 | 8.39 | 196.40 | 1318.49 | 414.74 | 1071.70 | 337.12 | 0.44 | 0.00 |
| 206 | 8.47 | 86.48 | 748.59 | 167.55 | 862.58 | 193.06 | 0.54 | 0.01 |
| 215 | 8.53 | 93.63 | 821.38 | 183.88 | 982.23 | 219.89 | 0.54 | 0.01 |
| 224 | 8.56 | 62.73 | 637.99 | 139.32 | 528.66 | 115.44 | 0.45 | 0.02 |
| 233 | 8.59 | 110.96 | 954.63 | 213.60 | 876.63 | 196.15 | 0.53 | 0.01 |
| 257 | 8.66 | 217.84 | 934.05 | 448.58 | 894.20 | 429.44 | 0.49 | 0.01 |
| 266 | 8.69 | 98.58 | 309.97 | 165.00 | 528.41 | 281.28 | 0.63 | 0.00 |
| 275 | 8.71 | 172.33 | 659.85 | 323.98 | 962.65 | 472.66 | 0.59 | 0.01 |
| 290 | 8.76 | 82.64 | 252.84 | 140.54 | 450.82 | 250.58 | 0.64 | 0.01 |
| 309 | 8.81 | 84.91 | 244.48 | 161.07 | 454.05 | 299.13 | 0.64 | 0.01 |
| 333 | 8.86 | 139.43 | 280.08 | 264.05 | 459.86 | 433.54 | 0.62 | 0.01 |
| 351 | 8.87 | 198.75 | 460.40 | 473.25 | 454.95 | 467.64 | 0.56 | 0.02 |
| 369 | 8.89 | 334.21 | 531.71 | 512.88 | 1089.11 | 1050.53 | 0.67 | 0.01 |
| 393 | 8.94 | 137.75 | 246.44 | 231.79 | 586.61 | 551.74 | 0.71 | 0.01 |
| 409 | 9.00 | 75.16 | 208.09 | 77.75 | 609.76 | 227.84 | 0.73 | 0.01 |
| 430 | 9.09 | 40.51 | 164.40 | 55.44 | 361.05 | 121.75 | 0.67 | 0.01 |
| 451 | 9.18 | 59.50 | 252.09 | 90.30 | 449.83 | 161.13 | 0.63 | 0.02 |
| 463 | 9.22 | 81.42 | 293.58 | 106.22 | 496.37 | 179.59 | 0.63 | 0.00 |
| 469 | 9.23 | 152.44 | 563.06 | 221.75 | 1075.33 | 423.49 | 0.66 | 0.00 |
| 484 | 9.29 | 46.88 | 264.30 | 100.42 | 242.54 | 92.16 | 0.46 | 0.02 |
| 506 | 9.37 | 118.63 | 552.66 | 235.73 | 594.99 | 253.79 | 0.56 | 0.02 |
| 515 | 9.40 | 169.77 | 596.78 | 260.54 | 1054.48 | 460.36 | 0.63 | 0.03 |
| 530 | 9.45 | 165.94 | 561.93 | 240.40 | 1146.97 | 490.68 | 0.66 | 0.01 |
| 554 | 9.55 | 73.39 | 288.39 | 111.16 | 638.02 | 245.93 | 0.69 | 0.00 |
